# The Limited Establishment of Native Ectomycorrhizal Fungi in Exotic *Eucalyptus* spp. Stands in Japan

**DOI:** 10.3389/fmicb.2021.597442

**Published:** 2021-03-18

**Authors:** Yoriko Sugiyama, Hirotoshi Sato

**Affiliations:** Graduate School of Human and Environmental Studies, Kyoto University, Kyoto, Japan

**Keywords:** ectomycorrhiza, distribution, eucalyptus, exotic host, host compatibility

## Abstract

Host specificity may potentially limit the distribution expansion of ectomycorrhizal (ECM) fungi into areas where their original host plants are absent. To test this hypothesis, we investigated whether populations of native ECM fungi may establish in stands of exotic host trees, namely those of the *Eucalyptus* species, in Japan. ECM fungal communities associated with eucalyptus and surrounding native host species (*Pinus thunbergii* and Fagaceae spp.) were investigated at two sites; one site in which eucalyptus and native trees were growing in isolation, and a second site in which these species were mixed. To identify fungal taxa, the nuclear ribosomal internal transcribed spacer region 1 was sequenced for the ECM fungi from the root tips and clustered into operational taxonomic units (OTUs). To confirm whether the retrieved OTUs were native to Japan, they were queried against the entire database of the National Center for Biotechnology Information, UNITE, and GlobalFungi, whereby sampling locations and associated hosts were obtained from sequences with ≥97% similarity. Eucalyptus trees were associated with seven and 12 ECM fungal OTUs, including putatively exotic OTUs in isolated and mixed sites, respectively. Among the 36 and 63 native ECM fungal OTUs detected from native hosts at isolated and mixed sites, only one OTU was shared with eucalyptus at the respective sites. This means that most native ECM fungi in Japan may be incapable of forming an association with exotic *Eucalyptus* spp. Notably, even ECM fungi associated with both *Pinus* and *Quercus* were not detected from eucalyptus, suggesting that host-fungus incompatibility is determined not only by host phylogenetic relatedness but also by host biogeographic affinities. Our findings show that the incompatibility with eucalyptus as well as dispersal limitation may prevent the distribution expansion of native ECM fungi in Japan into the distribution ranges of *Eucalyptus* spp., where the original hosts are absent.

## Introduction

Ectomycorrhizal (ECM) fungi form mutualistic associations with a variety of angiosperm and gymnosperm plant species (Smith and Read, 2008). Species belonging to approximately 40 angiosperm families, including Fagaceae, Betulaceae, Dipterocarpaceae, and Myrtaceae, are known to form ECM. Among the gymnosperms, the Pinaceae family is well-known as an ECM host lineage. It is estimated that a total of 8,500 flowering plant species are capable of forming associations with ECM fungi (Brundrett and Tedersoo, 2018). Nevertheless, due to host specificity, each ECM fungal species forms mutualistic associations with a limited range of host lineages within these ECM tree species (Molina et al., 1992; Molina and Horton, 2015). To date, host specificity in ECM fungi has been inferred in various studies (Molina and Trappe, 1994; Sato et al., 2007; Yamada et al., 2010).

Host specificity is a factor that is able to impact the distribution of ECM fungi. For example, Sato et al. (2012) estimated the potential distributions of ECM fungi collected from Yakushima Island, Japan with using the records in GenBank database, and assessed how the distribution ranges of ECM fungi are determined. The aforementioned study demonstrated that most ECM fungi could only be distributed in regions with ECM plant taxa that are similar to those on Yakushima Island, implying that the distribution ranges of ECM fungi are restricted by host specificity rather than by climate preference and dispersal limitations. Since the global distribution of ECM fungi has been rarely investigated (Sato et al., 2012; Tedersoo et al., 2014; García-Guzmán et al., 2017; Bazzicalupo et al., 2018), further studies are required to elucidate the influence of host specificity on the distribution ranges of ECM fungi.

If host specificity is a major factor determining the distribution ranges of ECM fungi, ECM fungi are expected to be incompatible with ECM tree species distributed outside their distribution ranges. To examine this hypothesis, it would be informative to investigate whether ECM fungi can establish associations with host species that have allopatric ranges of distribution; for instance, exotic plant species. Thus far, many ECM trees, such as *Pinus* spp., *Eucalyptus* spp., and *Acacia* spp., have been planted in regions beyond their natural ranges for various purposes (Richardson and Rejmánek, 2011). In these plantations, the richness of associated indigenous ECM fungi has been found to be low (Díez, 2005; Chen et al., 2007; Tedersoo et al., 2007; Nuñez et al., 2009; Dickie et al., 2010; Pennington et al., 2011). Based on this previous evidence, it can be hypothesized that few indigenous ECM fungi can infect exotic tree species due to incompatibility.

However, there is uncertainty as to what extent native ECM fungi are able to infect exotic host trees when the original host plants are absent. Previous studies are generally conducted in forests where native and exotic tree species grow together to a greater or lesser extent. Although some native ECM fungi have been reported to infect exotic host species in such mixed-stand forests (Bahram et al., 2013; Rudawska et al., 2018; Santolamazza-Carbone et al., 2019), caution should be exercised in interpreting these results, as the host ranges of ECM fungi could be broadened in areas where multiple host plants are growing together, owing to infection via common mycelial networks (Smith et al., 1995; Suz et al., 2017). If this hypothesis is true, it is unclear whether ECM fungi associated with exotic hosts in mixed forests are able to establish an association with novel host plant species in areas where their original host plants are absent. To test whether host specificity greatly influences the distribution of ECM fungi, it is necessary to investigate ECM fungal communities in areas populated exclusively by exotic host tree species.

In addition, to assess the aforementioned hypothesis, it would also be vital to compare the associated ECM fungi of exotic trees with those of surrounding native tree species. Some previous studies conducted in pure exotic plant forests have revealed a low diversity of native ECM fungi associated with exotic hosts (Chen et al., 2007; Pennington et al., 2011). However, it remains unclear whether the observed low diversity is due to the incompatibility between native fungi and exotic host species or to the particular characteristics of the study sites (Tedersoo et al., 2007). Thus, to gain a more specific insight into the effect of host specificity, comparisons of the ECM fungal communities associated with neighboring exotic and native hosts are required.

The present study aims to determine whether native ECM fungi is able to colonize exotic host species independently of infection via mycelial networks shared with native host species. To this end, we selected *Eucalyptus* spp. as the exotic hosts and examined whether the ECM fungi associated with native hosts were shared with eucalyptus trees in two plantation sites in Japan. Whilst trees in the *Eucalyptus* genus are naturally distributed in Australasia (Hermsen et al., 2012), they have never been part of the native flora of Japan, even in prehistoric times (Hermsen et al., 2012). At the main study site (Kobe Oji Zoo), eucalyptus trees are spatially isolated from other ECM hosts. As such, it is unlikely that infection occurs through mycelial networks shared between native and exotic host species. The barcode sequences of ECM fungi from the root tips were determined and subjected to the basic local alignment search tool (BLAST). The global distributions and host ranges of ECM fungi associated with eucalyptus trees and/or native host species were determined based on annotations of retrieved sequences. A similar survey was carried out at a site where eucalyptus trees were growing amongst native host species (the Botanical Gardens of Osaka City University). The hypothesis tested in this study is that most native ECM fungi are unable to establish populations in eucalyptus plantations, particularly when they are spatially isolated from native species.

## Materials and Methods

### Sampling Site

Sampling was conducted at two sites, namely, Kobe Oji Zoo (Kobe, Hyogo, Japan; hereafter Oji Zoo: 34°42′39″N, 135°12′54″E) and the Botanical Gardens of Osaka City University (Katano, Osaka, Japan; hereafter Botanical Gardens: 34°45′54″N, 135°40′51″E) (Table 1 and Supplementary Figure 1). Oji Zoo, which covers an area of 80,618 m^2^, is located in a residential area, and contains seven species of eucalyptus species planted between 5 and 30 years ago, which are maintained as food for koalas. These eucalyptus species were brought to Japan in the form of seeds from Australia, and were introduced to the zoo as seedlings, having initially been raised in an external nursery. Within the zoo, the trees were planted in several distinct locations (Supplementary Figure 1), at each of which, one to five species have been planted together. Along with these exotics, there are also native Japanese ECM tree species, namely, four *Quercus* spp. (*Q. glauca*, *Q. phillyraeoides*, *Q. myrsinifolia*, and *Q. acutissima*) and *Pinus thunbergii*. These native ECM tree species grow away from the eucalyptus trees (Supplementary Figure 1), and thus there is little likelihood of native ECM fungi infecting eucalyptus trees via common mycelial networks shared with native ECM tree species. In addition to these ECM tree species, several non-ECM tree species (including *Cerasus* × *yedoensis* and *Cinnamomum camphora*) also grow within the zoo premises.

**TABLE 1:** Description of the sampling sites.

Kobe Oji Zoo		The Botanical Gardens of Osaka City University
**Locality**		
Latitude	34°42′39″	34°45′54″
Longitude	135°12′54″	135°40′51″
**Host species sampled**		
Eucalyptus	*Eucalyptus tereticornis, E. punctata, E. camaldulensis, E. melliodora, E. microcorys, E. robusta, E. goniocalyx*	*Eucalyptus cypellocarpa, E. robusta, E. camaldulensis*
Native	*Quercus glauca, Q. phillyraeoides, Q. myrsinifolia, Q. acutissima, Pinus thunbergii*	*Quercus glauca, Lithocarpus glaber*
**Number of soil samples**		
Eucalyptus	52	4
Native	24	15

The Botanical Gardens of Osaka City University extend over an area of 255,300 m^2^ (including buildings within the gardens). The garden consists of several vegetation areas, among which Australia area is included. In an Australian area, 14 eucalyptus trees were introduced from Tennoji garden (Tennoji, Osaka, Japan) in 2006, and less than 10 now remain. No records are available to indicate the growth stage of the eucalyptus trees when initially introduced to Japan. Although there are no other exotic ECM host species growing within the vicinity of these eucalyptus individuals, some native ECM hosts (*Q. glauca* and *Lithocarpus glaber*) do grow nearby or mixed with eucalyptus. Outside the Australia area, Fagaceae species are found commonly throughout the gardens.

Given the small number of resident eucalyptus trees in the Botanical Gardens, greater emphasis was placed on data obtained from the Oji Zoo.

### Sampling and Sample Processing

At each site, soil samples (10 × 10 × 5 cm) containing fragments of the root system of the target host species were collected from beneath the target host species. When the collected soil sample only included a few fine roots, an additional root sample was collected from around the sampling points to obtain more root tips that were enough to check mycorrhizal colonization. In Oji Zoo, whether the obtained roots definitely belonged to the target species was not confirmed because trees belonging to different ECM host families were not growing close together. All samples were taken >3 m apart from one other.

At Oji Zoo, 52 eucalyptus and 24 native ECM tree samples were collected in July and December 2019, respectively. The eucalyptus samples were collected from five discrete sites within the zoo premises, each of which was planted with one to five eucalyptus species. However, as comparisons among eucalyptus species was not the purpose of the present study, we did not differentiate among the different species. Four out of five native tree species growing in the zoo were assessed as native ECM trees (Table 1). The breakdown of the 24 samples is as follows: 3 samples from beneath *Q. phillyraeoides* individuals, seven from *Q. glauca*, 12 from *Q. myrsinifolia*, and two from *P. thunbergii*.

In the Botanical Gardens, four eucalyptus samples and 15 Fagaceae samples were collected in July 2019. The Botanical Gardens records indicated that the sampled eucalyptus species were *E. cypellocarpa*, *E. camaldulensis*, and *E. robusta*. Among the 15 Fagaceae samples, three were from *Q. glauca* and one was from *L. glaber*, which was growing in close proximity to the eucalyptus trees. The remaining 11 samples were collected from under Fagaceae trees growing at least 50 m away from the eucalyptus species. Collected samples were maintained in plastic bags and frozen at −20°C until further use.

In the laboratory, each soil sample was slightly defrosted at room temperature (20–25°C). All fine roots were washed out with tap water. Soil particles and debris were carefully removed from the roots with forceps, and for samples collected from the Botanical Gardens, the roots were divided into different root systems. This is because these samples may have contained both Fagaceae and eucalyptus roots within the same sample. Then, more than 30 root tips (1-2 mm in length) per sample were collected with the aid of a Stemi DV4 stereo microscope (Carl Zeiss, Oberkochen, Germany) at ×20 magnification. At this point, all root tips showing evidence of ECM fungal colonization (i.e., changes in morphology or color) were sampled. In addition, root tips lacking these features were randomly sampled. For Oji Zoo samples, root tips contained within the same soil sample were pooled (i.e., resulted in 52 eucalyptus and 24 native host samples). For Botanical Gardens samples, root tips from the same root system were pooled to give a single sample, yielding a total 226 (27 eucalyptus and 199 native hosts) samples, which were stored in 70% ethanol (w/v) at −20°C until DNA extraction.

### DNA Extraction, Polymerase Chain Reaction Amplification, and Sequencing

Total DNA was extracted from root tips using DNeasy Plant mini kits (Qiagen, Hilden, Germany) following the manufacturer’s protocol. To analyze the fungal communities colonizing the roots, the ribosomal internal transcribed spacer regions (ITS1-5.8S-ITS2) of the ribosomal DNA were amplified using the ITS1F_KYO2 and ITS4_KYO2 (Toju et al., 2012) primer pair fused with an Illumina sequencing primer and six random bases (N). Polymerase chain reaction (PCR) was conducted using 10 μL of a KOD FX Neo (TOYOBO, Osaka, Japan) buffer system, containing 1 × PCR buffer, 0.4 mM deoxynucleoside triphosphates, 3 nmol each of the forward and reverse primers, 0.2 units of KOD FX Neo polymerase, and 1.0 μL of template DNA. The PCR conditions were as follows: an initial denaturation for 2 min at 94°C; followed by 40 cycles of 10 s at 98°C; 30 s at 60°C; 30 s at 68°C; and a final extension for 5 min at 68°C. To subsequently fuse the 8 bp identifier indices (Hamady et al., 2008) and the MiSeqP5/P7 adapter to the initial PCR amplicons, we conducted an additional PCR using the same PCR mixture and conditions as the initial PCR. The only change to the protocol was that the number of cycles had been reduced to 12. The resultant PCR amplicons were pooled and purified using AMPure XP (Beckman Coulter, Brea, CA, United States). The purified library was then excised using E-Gel SizeSelect (Thermo Fisher Scientific, Waltham, MA, United States). Among the 52 eucalyptus and 24 native samples from Oji Zoo, products had been successfully amplified from 39 and 23 samples, respectively. Among the 27 eucalyptus and 199 native samples collected from the Botanical Gardens, products were amplified from 21 and 178 samples, respectively.

For samples collected from the Botanical Gardens, host tree species identification was conducted simultaneously with fungi, based on the sequence of the ribulose-1, 5-bisphosphate carboxylase/oxygenase large subunit (*rbcL*) region. To amplify the *rbcL* region, we used the newly developed primer pair: 5′-TACTGCAGGTACATGYGAA-3′ as the forward primer, and 5′-ATATGCCAAACGTGAATACC-3′ as the reverse primer. The PCR mixture and program for the first and second PCRs were the same as those used to amplify the fungal DNA. The exception to this was that the annealing temperature was 55°C in the first round of PCR. The amplicon libraries were sequenced via 2 × 250-bp paired-end sequencing on the MiSeq platform (Illumina, San Diego, CA, United States) using MISEQ v.2 Reagent Nano Kits, following manufacturer’s instructions.

### Bioinformatics

MiSeq sequencing yielded 219 984, 433 496, and 638 791 reads for fungi from the Oji Zoo, Botanical Gardens, and plants of the Botanical Gardens, respectively. The retrieved reads were processed using CLAIDENT version 0.2.2018.05.29 (Tanabe and Toju, 2013). Reads were de-multiplexed using the “clsplitseq” command, and the resulting reads were deposited in the Sequence Read Archive of the DNA Data Bank of Japan (accession number: DRA010081). Using the “clfilterseq” command, we trimmed low-quality 3’ tails, and low-quality reads were filtered based on a minimum quality value of 30. For fungal reads, the resulting forward and reverse reads were merged using the “clconcatpair” command with the option mode = NON. This mode assumes that there is no overlap between forward and reverse reads, enabling us to concatenate reads in the following order: reverse reads, artificial sequence (ACGTACGTACGTACGT), and forward reads. For plant reads, forward and reverse reads were merged using the option mode = OVL. This mode assumes that there is an overlap between these reads. From the resulting merged reads, potentially noisy and chimeric sequences were eliminated using the “clcleanseqv” command. Remaining reads were clustered into operational taxonomic units (OTUs) with similarity thresholds of 0.97 and 1.00 for fungal ITS and plant *rbcL*, respectively, using the “clclassseqv” command. For OTU clustering of the fungal ITS reads, the artificial sequences were not taken into account. Potentially chimeric OTUs were eliminated using UCHIME (Edgar et al., 2011) without any references.

After removing artificial sequences, OTUs were divided into forward (ITS1) and reverse (ITS2) sequences. The taxonomic assignment of these regions was conducted separately using CLAIDENT. Among the taxonomic assignment results for ITS1 and ITS2, the lower level taxonomic assignment was accepted. In this procedure, OTUs with identifications showing clear differences between ITS1 and ITS2 (e.g., contradiction of taxonomic assignment at the phylum level), were regarded as chimeric and discarded. In addition, in a sample × OTU matrix, cells with a read number of one were converted to zero to reduce the effects of tag jumps and contamination. After taxonomic assignment with CLAIDENT, we referred to the UNITE database (Nilsson et al., 2019) to determine the taxonomies sequences that could not be assigned to any families or genera using CLAIDENT. Supplementary Table 1 presents the results of the CLAIDENT, UNITE search, and the number of reads for OTUs. The functional groups of the obtained OTUs were inferred using FUNGuild (Nguyen et al., 2016) based on their taxonomy (Supplementary Table 1). Further, for OTUs that were assigned to several functional groups, including ECM (e.g., those identified as Ectomycorrhizal-Fungal Parasite), we referred to reviews by Tedersoo et al. (2010) and Tedersoo and Smith (2013) to verify whether the genus or family of the OTU is included in the list. The resulting ECM fungal OTUs (ECM OTUs) were used in further analyses (see Supplementary Table 1). For the reads of plant samples obtained from the Botanical Gardens, in order to eliminate potentially contaminated or error reads, OTUs with a read number that was less than 10% of the total read number were eliminated from each sample. The families of the remaining OTUs were then recorded to distinguish whether the host species was a *Eucalyptus* or a native Fagaceae species. All remaining OTUs were assigned to either *Eucalyptus* or Fagaceae. As one sample contained both Fagaceae and Myrtaceae OTUs, it was eliminated from further analyses.

### Data Analysis

To compare the ECM fungal colonization rate between the native and eucalyptus host species, Fisher’s exact test was conducted in R version 3.6.0 (R Core Team, 2018). In this analysis, the colonization rate was assessed based on the soil sample, and calculated by dividing the number of sequenced soil samples by the number of soil samples from which ECM fungi were detected.

Sample-based OTU accumulation (rarefaction) curves were depicted separately for the ECM fungal communities associated with eucalyptus and native hosts using the “iNEXT” package in R with 1,000 replications. Rarefaction curves for native host species were constructed for two datasets; including and excluding *P. thunbergii* samples. This was done as phylogenetically distant hosts often harbor distinct ECM fungal communities (Ishida et al., 2007; Matsuoka et al., 2020), which may affect ECM fungal OTU richness. As the number of samples obtained from the Botanical Gardens was relatively small, these statistical analyses were conducted only on samples collected from Oji Zoo.

To identify the potential origins and host range of detected ECM fungal OTUs, we examined the geographic distribution and host range of each OTU. To achieve this, 100 BLAST-hit sequences were downloaded from the NCBI database, and a p-distance matrix between the focal OTU and retrieved sequences was calculated using MEGA 7 (Kumar et al., 2016). It was assumed that sequences with ≥97% similarity in terms of p-distance were derived from the same species. The sampling locations and associated hosts of these sequences were retrieved from the annotation information within the NCBI database. The original publication was consulted in cases where the retrieved country and/or host were not specified in the annotation. The same analyses were conducted on the UNITE and GlobalFungi (Větrovský et al., 2020) databases. In the UNITE search, only sequences without GenBank accession numbers were downloaded. In these evaluations, only sequences retrieved from roots or sporocarps were included, and sequences from soil were removed to exclude the possibility that focal fungus spores had been dispersed at the sampling location without the fungus actually being established.

## Results

### The ECM Fungal Community at Oji Zoo

**FIGURE 1:**
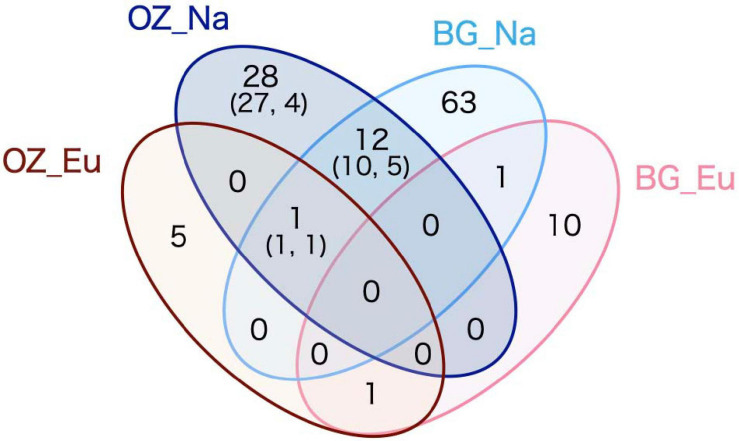
Venn diagram depicting the retrieved operational taxonomic unit (OTU) numbers. OZ and BG denote Oji Zoo and Botanical Gardens, respectively, and Eu and Na denote eucalyptus and native hosts, respectively. The numbers within parentheses are the number of ECM fungal OTUs detected from Fagaceae (former) and *Pinus thunbergii* (latter) samples.

After filtering and cleaning, there were remaining 52,705 reads from the Oji Zoo samples; these were clustered into 456 OTUs, of which 47 were assigned to ECM fungal taxa (Supplementary Table 1). ECM fungal OTUs were detected in all 23 native host tree samples (i.e., *Quercus* and *Pinus* samples), and only in 10 of 39 eucalyptus samples. The rate of fungal infection significantly differed between native host and eucalyptus samples (Fisher’s exact test, *p* < 0.001). Among the 41 OTUs retrieved from native host samples, 10 were from *P. thunbergii*, 38 were from *Quercus* spp., and seven were shared between *Pinus* and *Quercus* host species. In contrast, only seven ECM fungal OTUs were retrieved from eucalyptus species. One OTU was shared between native and eucalyptus samples (Figure 1), detected in *P. thunbergii* and *Quercus* spp. The rarefaction curve for eucalyptus samples almost reached an asymptote, in contrast to that of the native host species, irrespective of whether *P. thunbergii* samples had been included. This indicates the presence of more ECM fungal OTUs associated with native hosts (Supplementary Figure 2).

Taxonomically, among the 41 ECM fungal OTUs retrieved from native host samples, 11 belonged to Ascomycota and 30 belonged to Basidiomycota. These 41 OTUs were classified into 10 families. Although Thelephoraceae was the most OTU-rich family associated with native host species (17 OTUs, 41.5% of the total number of ECM fungal OTUs), Thelephoraceae OTUs did not dominate in eucalyptus root samples. The seven OTUs retrieved from the eucalyptus samples were identified as *Cenococcum geophilum* (3), *Laccaria* sp. (1), *Tomentella* sp. (1), and *Scleroderma albidum* (2). Of these, the OTU shared with the native host species was *C. geophilum*.

### Host Plants and ECM Fungal Community in the Botanical Gardens

All root systems from the four soil samples collected from around the eucalyptus trees belonged to the eucalyptus species, whilst those obtained from the 15 soil samples collected from around native Fagaceae plants belonged to the Fagaceae species. The exception to this was a single sample that contained both eucalyptus and Fagaceae OTUs, and thus was not used in subsequent analyses. Ectomycorrhizal fungal OTUs were detected in all samples collected from the vicinities of native Fagaceae and eucalyptus trees.

The 247 114 reads obtained for fungal ITS were clustered into 678 OTUs, of which 86 were assigned to ECM fungal taxa. Seventy-five ECM fungal OTUs were retrieved from 15 Fagaceae samples, classified into 14 families, among which Thelephoraceae (20 OTUs, 26.7%) and Russulaceae (17 OTUs, 22.7%) were the most OTU-rich families. A comparatively smaller number of ECM fungal OTUs (12) were obtained from four eucalyptus samples, one of which was also detected in native host samples (Figure 1). The 12 ECM fungal OTUs retrieved from the eucalyptus samples were identified as *Laccaria* spp. (4), Thelephoraceae (3), *Tomentella* spp., *Tuber* spp. (2 each), and *Sebacina* sp. (1). Among these OTUs, one of the *Tomentella* OTUs was also detected in the native host samples. Furthermore, of the three *Laccaria* OTUs, one was also detected in the eucalyptus samples obtained from Oji Zoo.

### Sampling Localities and Host Species Identified Based on BLAST-Hit Sequences

Tables 2, 3 and Supplementary Table 2 present the sampling localities and host species of the GenBank, UNITE, and GlobalFungi sequences that showed ≥97% similarity with ECM fungal OTUs detected in the present study (hereafter, BLAST-hit sequences). Among the 47 and 86 ECM fungal OTUs from Oji Zoo and the Botanical Gardens, eight, and six OTUs, respectively, did not show ≥97% similarity with any reference sequence. Among the ECM fungal OTUs from Oji Zoo and the Botanical Gardens that did show ≥97% similarity with reference sequences, we were unable to verify sampling localities for nine and ten OTUs, respectively. Accordingly, sampling localities and host species were identified for 30 and 70 OTUs, respectively (Supplementary Table 2).

**TABLE 2:** List of ECM fungi detected from eucalyptus, along with the presumed distribution and hosts.

OTU_ID: Species hypothesis ID^a^ taxa	Site^b^	Eucalyptus Forests	Forests of other trees^c^
OTU_0685: SH1553003.08FU *Laccaria*	OZ, BG	Australia, Brazil, Cameroon, Kenya, Madagascar, Spain, Italy	Argentina (*Nothofagus*/*Carpinus*), New Zealand (*Leptospermum*/*Nohofagus*), Australia (*Allocasuarina*^∗^/*Nothofagus*^∗^/*Leptospermum*^∗^/*Acacia^∗^)*
OTU_0488: SH1544059.08FU *Scleroderma albidum*	OZ		Argentina (*Ilex*), Cameroon (*Fabaceae*), China, North America
OTU_0692: SH1639590.08FU *Cenococcumgeophilum*	OZ		China (*Epipactis*), USA (*Quercus*)
OTU_0710: SH1639590.08FU *C.geophilum*	**OZ**		New Zealand (*Nothofagus*), Japan (*Pinus)*, China (Fagaceae/Pinaceae/*Polygonum*/*Kobresia*), USA (*Pyrola*/Fagaceae/Betulaceae*^∗^*/*Salix*^∗^/Pinaceae*^∗^*), Canada (Pinaceae/*Quercus*/*Poplus*), Germany (*Alnus*)
OTU_0746: No SH match *Tomentella*	OZ	No other record.	
OTU_0764: SH1639590.08FU *C. geophilum*	OZ		Japan (*Quercus*^∗^/*Pinus*^∗^/*Ilex*^∗^)
OTU_0770: SH1544059.08FU *S. albidum*	OZ	Australia, Brazil, Cameroon, Portugal China, India, Pakistan, Senegal	Cameroon (Fabaceae*^∗^*), Cape Verde, Australia, Argentina (Ilex), New Caledonia (*Acacia*), Estonia (Salix/Populus)
OTU_0381: SH1553011.08FU *Laccarialateritia*	BG	No information available on the distribution and the host.	
OTU_0416: SH1502458.08FU *C.geophilum*	BG	No information available on the distribution and the host.	
OTU_0689: SH1553011.08FU *Laccaria*	BG	Australia, Cameroon, Spain, Sweden, UK	Australia (*Nothofagus*/*Acacia*^∗^), New Zealand (*Nothofagus*/*Leptospermum)*, Colombia (Acacia) Germany (*Populus*), Netherland (*Corylus*^∗^), Canada
OTU_0747: SH1502690.08FU *Tomentella*	BG		Germany (*Fagus*), Japan (Fagaceae*^∗^*/*Pinus*^∗^)
OTU_0751: SH1502458.08FU *Tomentella*	**BG**		China (*Quercus*), Japan (*Pinus*/*Goodyera*)
OTU_0753: SH1553011.08FU *Laccaria*	BG		Sweden (*Salix*)
OTU_0759: SH3593769.08FU *Tuber*	BG		Italy, USA (*Quercus*/*Malus*), Mexico
OTU_0767: SH3593769.08FU *Tuber*	BG	No other record.	
OTU_0784: No SH match *Sebacina*	BG		France *Erica*,China (*Castanopsis*/*Pinus*/*Rhododendron*), USA Ericaceae
OTU_0790: SH1503527.08FU Thelephoraceae	BG		Japan (Fagaceae/*Pinus*)
OTU_0791: SH1522923.08FU Thelephoraceae	BG		China (*Castanopsis*/*Pinus*/*Rhododendron*)

*^a^Species Hypothesis ID of best hit sequence in UNITE. ^b^OZ: Oji Zoo, BG: Botanical Garden. Broad letters indicate that the OTU was also detected in the native host samples at that site. ^c^Country and the associated host species in that countries of the similar sequences (≥ 97%) are shown. If the similar sequences were detected for the host species of different genera within the same family in a specific country, the family names are indicated instead of listing the genus. ^∗^The associations are uncertain (e.g., samples were collected in a mixed forests).*

**TABLE 3:**
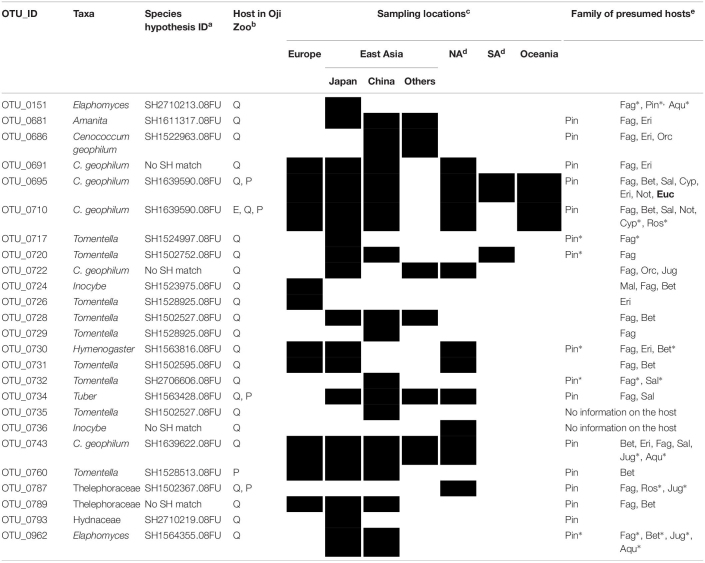
List of ECM fungi detected from native hosts in Oji Zoo, along with presumed distribution and hosts.

*Only those OTUs whose distribution could be assessed are shown. Black cells indicate that the similar sequences (≥97%) were detected in that region. For details on the collected countries and associated hosts, please refer to Supplementary Table 2. ^a^Species Hypothesis ID of best hit sequence in UNITE. ^b^E, F, and P indicate Eucalyptus spp., Quercus spp., and P. thunbergii, respectively. ^c^No BLAST-hit sequences were obtained from Russia, Asia outside of East Asia, or Africa. ^d^NA: North Americca, SA: South America. ^e^Pin: Pinaceae, Aqu: Aquifoliaceae, Bet: Betulaceae, Cyp: Cyperaceae, Eri: Ericaceae, Euc: Eucalyptus, Fag: Fagaceae, Jug: Juglandaceae, Mal: Malvaceae, Orc: Orchidaceae, Ros: Rosaceae, Sal: Salicaceae, Not: Nothofagus. ^∗^The association was uncertain (e.g., root samples were collected in a mixed-forests).*

Among the seven OTUs detected from Oji Zoo eucalyptus samples, we were unable to trace the sampling localities of the BLAST-hit sequences for one OTU (OTU_0746: *Tomentella* sp.). The sampling localities of the BLAST-hit sequences for OTU_0685 (*Laccaria* sp.) and OTU_0770 (*Scleroderma albidum*) were forests in Australia and other countries in the Southern Hemisphere outside the natural distribution of eucalyptus (e.g., Argentina, Cameroon, and New Zealand), along with eucalyptus plantations in other regions (Table 2). However, the BLAST-hit sequences for OTU_0770 included two sequences from Salicaceae forests in Estonia (Table 2). The BLAST-hit sequences of the other *Scleroderma albidum* OTU (OTU_0488) were from the Northern (North America and China) and Southern (Cameroon and Argentina) Hemispheres. The sampling localities of the three *C. geophilum* OTUs were largely from Japan, China, and North America, with some from Germany and New Zealand.

Among the 12 OTUs detected from the Botanical Gardens eucalyptus samples, we were unable to identify similar sequences for OTU_0767 (*Tuber* sp.), and were unable to trace the sampling localities for OTU_0381 (*Laccaria lateritia*) and OTU_0416 (Thelephoraceae sp.). For one of the three *Laccaria* OTUs (OTU_0689) not detected in Oji Zoo samples, the fungal materials of the BLAST-hit sequences were identified as originating from natural *Eucalyptus* and *Nothofagus* forests (e.g., Australia and New Zealand), and forests not containing trees in these genera (e.g., Germany and Netherlands). The BLAST-hit sequences for the remaining seven OTUs were reported to originate only from the natural forests of the Northern Hemisphere (mainly China and Japan), and not from eucalyptus forests.

On the other hand, the BLAST-hit sequences for the OTUs detected from native host samples in the study sites were mainly reported as being from the apparently natural forests of the Northern Hemisphere (Table 3). Hosts for these sequences were reported to be those common to these regions, such as plants of the Fagaceae, Pinaceae, Betulaceae, Salicaceae, Juglandaceae, or Orchidaceae families (Table 3 and Supplementary Table 2). The accession numbers, p-distances to our sequences, sampling locations, associated hosts, and isolation sources for each BLAST-hit sequence are provided in the Appendix. For OTU_0710 (*C. geophilum* from an Oji Zoo eucalyptus sample), for which BLAST-hit sequences were obtained from both the Northern (e.g., Japan, China, United States, and Germany) and Southern (New Zealand) Hemisphere, all the BLAST-hit sequences from New Zealand differed by 2.99% from that of OTU_0710. Accordingly, when the similarity threshold was increased, all the BLAST-hit sequences for OTU_0710 were from the Northern Hemisphere. Apart from this, we found that increasing the similarity threshold did not significantly alter the BLAST results obtained.

## Discussion

In our sites, most of the native ECM fungi seem to be restricted from colonizing eucalyptus trees especially when infection through common mycelial networks is not possible. This appears to be true even for ECM fungi that can associate with both native angiosperms and gymnosperms. These findings support the idea that host-fungus incompatibility is one of the factors that could restrict ECM fungi from expanding their distribution ranges.

The results obtained for Oji Zoo were consistent with the hypothesis that host-fungus incompatibility inhibits the colonization of exotic host species by native ECM fungi. The geographical information obtained from BLAST-hit sequences indicates that the ECM fungal OTUs detected in native host samples could be native to Japan and that they are hardly associated with exotic eucalyptus trees in Oji Zoo (Figure 1 and Tables 2, 3). Given that native hosts were found to harbor many ECM fungal OTUs, it would appear unlikely that a shortage of inocula is the reason the ECM fungi of native hosts did not colonize eucalyptus plants. Moreover, given that we detected a low colonization rate in eucalyptus samples, it seems equally unlikely that competition (Wu et al., 1999; Smith et al., 2018) and/or priority effect (Kennedy et al., 2009; Fukami, 2015) prevent native ECM fungi from colonizing eucalyptus trees. We accordingly consider it more probable that the observed restricted colonization is attributable to host–fungus incompatibility. This assumption would appear to be corroborated by the finding that the ECM fungal species that are putatively identical to those detected only in the native *Quercus* and *Pinus* samples are found only in those regions where Fagaceae and Pinaceae are distributed (i.e., Europe, Asia, and North America; Alfaro Reyna et al., 2018). Although it should be highlighted that, in the Southern Hemisphere, there have been comparatively few relevant studies and that the numbers of deposited sequences are currently relatively small (Dickie and Moyersoen, 2008), the results of the present study is consistent with the opinion of Sato et al. (2012) that host-fungus incompatibility limits the distribution range of ECM fungi.

The Oji Zoo results also suggest the possibility that ECM fungi associated with both angiosperm and gymnosperm hosts are incapable of colonizing eucalyptus trees. Some of the native ECM fungal OTUs identified in the present study (e.g., OTU_0704, 0734, and 0787) were detected in both gymnosperms (*P. thunbergii*) and angiosperms (*Quercus* spp.), indicating that these OTUs can be categorized as broad host range fungi, according to the conventional definition (Molina et al., 1992). Moreover, among the OTUs that were retrieved from either *P. thunbergii* or *Quercus* spp. samples in Oji Zoo, several OTUs are suspected to be associated with both angiosperm and gymnosperm hosts based on an analysis of their BLAST-hit sequences. However, these OTUs were not detected in the root systems of eucalyptus trees in Oji Zoo or elsewhere in the world (Table 3), thereby indicating that even broad host range OTUs may be unable to establish associations with exotic host species. To the best of our knowledge, there have been no previous studies that have tackled the incompatibility between broad host range ECM fungi and exotic hosts. This possibility has, nevertheless, been proposed by Díez (2005), who surveyed the ECM fungal community in a Spanish eucalyptus plantation, although did not assess the host ranges of the native ECM fungi. The findings presented herein provide evidence in support of the hypothesis put forward in Díez (2005), and thereby indicate that host plants that are compatible with ECM fungi are not only determined by phylogenetic relatedness but also by the similarity of their biogeographic background.

*Cenococcum geophilum* seems to be an exceptional ECM fungal species that is associated with phylogenetically and biogeographically distinct host plants. Although it should be cautioned that *C. geophilum* is considered to be a species complex (Douhan and Rizzo, 2005; Obase et al., 2016), some OTUs of *C. geophilum* are shared among continents or host taxa (Ishida et al., 2007; García-Guzmán et al., 2017), indicating that the complex comprises multiple, widespread generalist fungal species. In the present study, we confirmed that a *C. geophilum* OTU (OTU_0710) had colonized both native and eucalyptus trees in Oji Zoo, under circumstance in which infection through common mycelial networks was unlikely to occur. Furthermore, based on our analysis of BLAST-hit sequences, the same OTU appears to be distributed in a broad swathe of regions, including East Asia, Europe, North America, and Australasia, and can establish mycorrhizal associations with a wide range of host species, including *Nothofagus*, Betulaceae, Fagaceae, and Pinaceae species. However, given that the OTU_0710 BLAST-hit sequence from *Nothofagus* forests in New Zealand differed by almost 3% from the sequence determined in the present study, these findings should be interpreted with caution (Schoch et al., 2012). Nevertheless, collectively, the evidence to date supports the notion that *C. geophilum* could be distinguished from other ECM fungal species in having an extremely broad host compatibility and distribution range.

Most ECM fungi associated with exotic eucalyptus trees also appeared to be exotic in Japan and originated from those associated with *Eucalyptus* spp. and other host plants in the Southern Hemisphere, in line with reports from previous studies (Díez, 2005; Pennington et al., 2011). The analysis of BLAST-hit sequences indicated that two OTUs detected from eucalyptus samples in Oji Zoo (OTU_0685, *Laccaria* sp. and OTU_0770, *Scleroderma albidum*) originated from the Southern Hemisphere (Australia, New Zealand, Argentina, or New Caledonia) and eucalyptus plantations. Although eucalyptus species are not native to New Zealand, Argentina, or New Caledonia, the native flora in these countries include ECM host genera that are also native to Australia, such as *Nothofagus* and *Leptospermum*. Thus, it is possible that these fungi may be able to establish associations with eucalyptus species and other host species in their original distribution. Indeed, OTU_0685 is presumed to be con-specific to “Trappe14544”; this is an ECM fungus reported to be associated with *Nothofagus* and *Eucalyptus* (Wilson et al., 2017). For OTU_0448 (*S. albidum*), *S. albidum* is widely known as an introduced species from the Southern Hemisphere, associated with *Eucalyptus* (Guzmán et al., 2013; Sulzbacher et al., 2018). Thus, although none of the BLAST-hit sequences were from eucalyptus forests, it is possible that the distribution of these BLAST-hit sequences does not reflect the original distribution of OTU_0448. Although the distribution of one OTU (*Tomentella* sp.) remains unknown, our results indicate that ECM fungi native to Japan are not among the main taxa comprising the ECM fungal community associated with eucalyptus in Oji Zoo. Overall, strong host-fungus incompatibility seems to force exotic eucalyptus trees in Oji Zoo to associate only with ECM fungi growing in their original distribution, although the infection route remains unclear (e.g., long-distance dispersal and/or infection in Japanese eucalyptus nurseries).

The findings of the present study have important implications with respect to our understanding of the biogeography of ECM fungi. Peay et al. (2012) have previously shown that, as the distance from the spore source increases, the number of spores reach and the consequent probability of ECM fungal colonization declines, suggesting that the dispersal limitation is a crucial factor to determine the distribution of ECM fungi. Nevertheless, our results show host-fungus incompatibility between exotic host plant and native ECM fungi growing in the same area (within ca. 250 m), suggesting that native ECM fungi detected in our site are not easily established in novel areas where the original host plants are absent, even if a large number of spores reached the region.

Although the number of samples was small, the results obtained for the Botanical Gardens provide clues with respect to determining the conditions under which host–fungus incompatibility might be relaxed. Our findings for both the Botanical Gardens and Oji Zoo were largely comparable, in that we detected a lower ECM fungal OTU richness in eucalyptus samples than in surrounding native host samples, and that only a few OTUs were shared among eucalyptus and native host samples. However, there were also some notable differences in the results obtained from the two sites. In particular, we observed a higher colonization rate in the Botanical Gardens, although statistical significance was not tested. Moreover, based on our BLAST-hit sequence analysis (Table 2), some ECM fungal OTUs in eucalyptus samples collected from the Botanical Gardens (*Tomentella* spp., Thelephoraceae spp., *Sebacina* sp., and *Laccaria* sp.) were seemingly native to Japan. The BLAST-hit sequences also indicated that these OTUs are associated with a range of host species (e.g., Pinaceae, Betulaceae, and Fagaceae) that are distributed in the Northern Hemisphere, specifically in Japan and the surrounding countries (Table 2). We presume that differences in the results obtained for Oji Zoo and the Botanical Gardens might be attributed to the presence/absence of infection via mycelial networks shared between eucalyptus and native hosts, given that eucalyptus and native hosts grow in close proximity in the Botanical Gardens, but not in Oji Zoo. High infection rates in exotic hosts, as well as infection of exotic hosts by native ECM fungi, have also been reported previously in mixed forests composed of both native and exotic hosts (Jairus et al., 2011; Pennington et al., 2011; Moeller et al., 2015; Rudawska et al., 2018; Santolamazza-Carbone et al., 2019). Especially, Santolamazza-Carbone et al. (2019) reported lower infection rates and ECM fungal richness of eucalyptus growing inside the eucalyptus plantations than inside the natural forests, which is in line with our results. Though, in this previous study, indigenous trees were also growing inside the eucalyptus plantations, the low abundance of indigenous trees in these plantations may have made the successive formation of common mycelial networks between native and eucalyptus trees difficult. These results may also support the hypothesis that host-fungus incompatibility could be relaxed by common mycelial networks.

At last, there are some limitations in our study which impose us careful interpretation of the results. First, sampling was conducted at only two sites and there was no replication of either the mixed or isolated eucalyptus stands, which impedes generalizing these results. To confirm generality, it is necessary to conduct similar surveys in different areas and/or with a focus on different exotic host plant species. More empirical studies are also required to confirm whether native ECM fungi are capable of colonizing exotic host trees isolated from native ECM hosts. Second, in Oji Zoo, we collected native and eucalyptus samples in different months. Thus we cannot exclude the possibility that detected difference in ECM fungi between native and exotic hosts may be attributable to seasonal differences. However, the seasonal effect may be small as marked temporal changes in OTU richness and/or community members within the same year have never previously been observed (Koide et al., 2007; Courty et al., 2008; Sugiyama et al., 2020). Further studies addressing these issues would make a substantial contribution to the collective understanding of how host-fungus incompatibility may determine the biogeography of ECM fungi.

## Conclusion

This study found that eucalyptus species planted at selected study sites in Japan were associated with exotic ECM fungi that may have the same geographic origins as the exotic eucalyptus trees, while the surrounding native host species formed associations with the native fungi. These results indicate that most ECM fungi detected in the studied sites were incapable of associating with host species originating from regions outside of the potential distribution of the fungi themselves. Our results suggest that ECM fungi observed in the present study do not readily expand their distribution via host shifts to exotic host trees, such as eucalyptus, even if they have dispersed spores into the areas of the Southern Hemisphere. Notably, even ECM fungi associated with both angiosperm (e.g., *Quercus* spp.) and gymnosperm (e.g., *P. thunbergii*) hosts at the study sites did not appear to readily infect *Eucalyptus* species. The findings of the present study suggest the possibility that host-fungus incompatibility is determined not only by the phylogenetic relatedness of host plants but also by the past and present distribution of host plants. Future studies dealing with hosts other than eucalyptus in regions other than our sites would help us understand more fully the relatedness between ECM fungal-host specificity and distribution.

## Data Availability Statement

The datasets generated in this study can be found in the DDBJ Sequence Read Archive (DRA) under the accession number DRA010081 or BioProject Accession: PRJDB9656.

## Author Contributions

YS designed the study, performed the molecular analyses, and wrote the initial draft of the manuscript. YS and HS carried out the field work, analyzed the data, and interpreted the results. HS critically reviewed the manuscript. Both authors contributed to the article and approved the submitted version.

## Conflict of Interest

The authors declare that the research was conducted in the absence of any commercial or financial relationships that could be construed as a potential conflict of interest.

## Supplementary Material

Supplementary Figure 1 The locations of the sampling sites and the map of sampling points in Oji Zoo. In the map of Oji Zoo, the areas indicated in pink are eucalyptus stands, and those in blue are native host species. Within the map, the ID of samples taken from the stands is also indicated, which is consistent with the sample ID indicated in Supplementary Table 1.

Supplementary Figure 2 Sample size-based species accumulation (solid line) and extrapolation (dotted line) curves with 95% confidence intervals (shaded areas) for the ECM fungal OTU richness of eucalyptus (red) and native (blue) host samples in Oji Zoo. As native host samples, both Fagaceae and *Pinus thunbergii* samples (i.e., 23 samples) are included in **(a)** and only Fagaceae samples (i.e., 21 samples) are included in **(b)**.

Supplementary Table 1 The list of obtained operational taxonomic units (OTUs). OTUs denoted by the “T” in the column, “ECM,” are the putative ectomycorrhizal fungal OTUs based on the taxonomy implemented by CLAIDENT. OTUs denoted by the “T” in the column, “ECM_UNITE,” are the putative ectomycorrhizal fungal OTUs based on the taxonomy implemented by the CLAIDENT and UNITE searches, and were used for the analyses.

Supplementary Table 2 The distribution and the associated hosts of the similar sequences. If several countries are listed as collection locations, the country names are listed in parentheses. If similar sequences were detected from host species in different genera within the same family in a specific country, the family names are indicated instead of listing the genera. For samples collected in mixed forests and for which exact hosts were uncertain, the host taxa are indicated in parentheses.^∗^E: *Eucalyptus* spp., F: Fagaceae spp., P: *Pinus thunbergii.*

Supplementary Appendix The results of GenBank, UNITE, and GlobalFungi searches of all ECM fungi retrieved in this study. For each BLAST-hit sequence, the p-distance to the query OTU, the collection site (country), associated host, and sample type are also indicated. Sequences differing by more than 3% from those detected in this study are denoted by gray letters. Blank cells indicated that we were unable to obtain relevant information. In each seat, the results of the GenBank search are shown in rows without highlights, whilst the results of UNITE and GlobalFungi searches are shown in rows highlighted in yellow and blue, respectively. In UNITE search, sequences that have GenBank accession number or derived from soil are exclude.
