# Supplementary material for: The Limited Establishment of Native Ectomycorrhizal Fungi in Exotic Eucalyptus spp. Stands in Japan
Source: Front Microbiol. 2021 Mar 18;12:597442. doi: 10.3389/fmicb.2021.597442 (PMC8012522; doi:10.3389/fmicb.2021.597442)
Supplement: Supplementary Figure 1 — The locations of the sampling sites and the map of sampling points in Oji Zoo. In the map of Oji Zoo, the areas indicated in pink are eucalyptus stands, and those in blue are native host species. Within the map, the ID of samples taken from the stands is also indicated, which is consistent with the sample ID indicated in Supplementary Table 1. [file Data_Sheet_2.docx]

Supplementary Materials

**
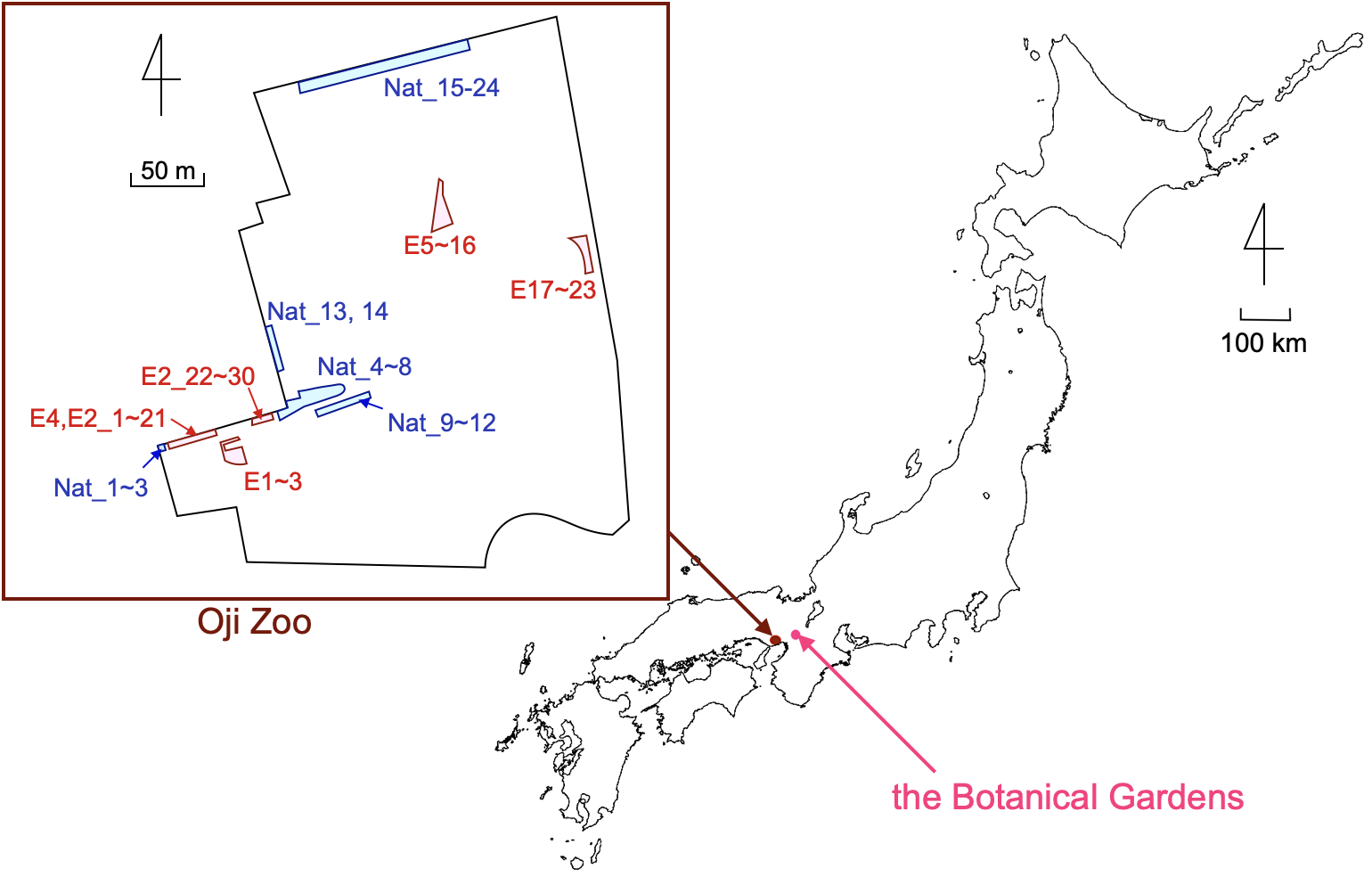
**

**Fig. S1**

The locations of the sampling sites and the map of sampling points in Oji Zoo.

In the map of Oji Zoo, the areas indicated in pink are eucalyptus stands, and those in blue are native host species. Within the map, the IDs of samples taken from the stands is also indicated, which is consistent with the sample ID indicated in Table S1. The precise map of sampling points within the Botanical Gardens is not included, as in the Botanical Gardens, *Eucalyptus* samples were taken from a single location.

**
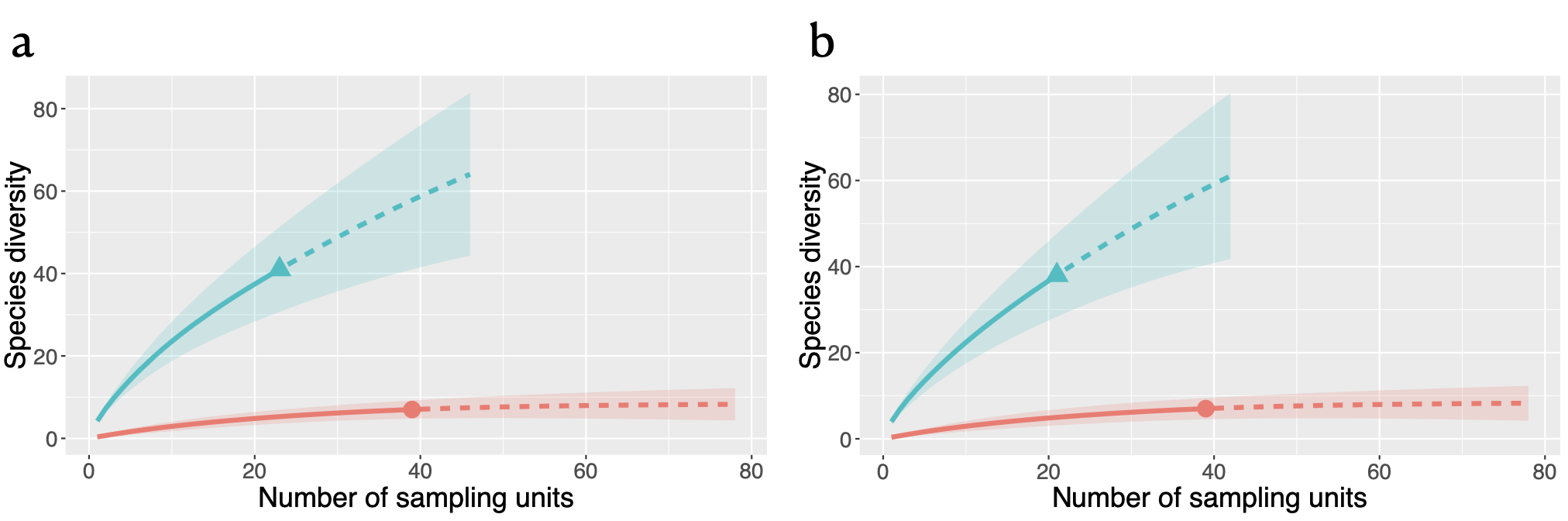
**

**Fig. S2**

Sample size-based species accumulation (solid line) and extrapolation (dotted line) curves with 95% confidence intervals (shaded areas) for the ECM fungal OTU richness of eucalyptus (red) and native (blue) host samples in Oji Zoo. As native host samples, both Fagaceae and *Pinus thunbergii* samples (i.e., 23 samples) are included in (a) and only Fagaceae samples (i.e., 21 samples) are included in (b)
